# Women’s and male partners’ socio-demographic and economic characteristics associated with contraceptive decision making in Nigeria

**DOI:** 10.1186/s12905-022-02045-w

**Published:** 2022-11-16

**Authors:** David Aduragbemi Okunlola

**Affiliations:** 1 Department of Sociology, College of Social Sciences and Public Policy, Florida State University, Tallahassee, USA; 2 Viable Knowledge Masters, Abuja, Nigeria

**Keywords:** Contraceptive decision-making, Partnered women, Nigeria

## Abstract

**Background:**

Women’s ability to make contraceptive decision can determine their contraceptive use which can improve their reproductive health and career. Improvement in such ability can increase contraceptive prevalence in Nigeria. However, factors that promote contraceptive decision-making among women are scarcely studied. This study examined factors associated with women’s individual or joint contraceptive decision-making in Nigeria.

**Methods:**

Secondary (cross-sectional) data were analysed. The data were extracted from the individual recode file of the 2018 Nigeria Demographic and Health Survey (DHS). Partnered women (i.e., currently married or living with a partner) aged 15–49 years and currently using contraceptives before the survey were considered. They constituted 4,823 in total. Their data were analysed using frequency and percentage distributions of variables, Chi-square tests of independence and multinominal logistic regression.

**Results:**

Findings reveal that 23% (1,125) of women made their own contraceptive decision, nearly 67% (3,213) were joint decision makers, and 10% (491) stated that their male partners had decided for them. The probability of solely making contraceptive decision and being a joint decision maker (relative to being a male partner’s decision) was higher among women above 29 years and aged 30–34 years (than women aged 15–24 years) respectively as well as among the employed (than the unemployed) and among those from Yoruba ethnic group (than their counterparts from Hausa/Fulani/Kanuri/Beri Beri) respectively. The probability of being responsible for contraceptive decision (than being the male partner’s decision) was higher among women from the Igbo group and women whose male partners desired more children (than those with the same number of desired children) respectively. The probability of being the main decision maker (relative to being the male partner) was lower among women in the poorer (RRR = 0.39; 95%CI = 0.21–0.73; *p* = 0.01), middle (RRR = 0.47; 95%CI = 0.25–0.90; *p* = 0.02) and richest (RRR = 0.41; 95%CI = 0.20–0.82; *p* = 0.01) groups respectively, than the poorest women. The probability of being a joint decision maker was higher among women with secondary education (than the uneducated), practised Christianity (than the Muslims/ others), and among those residing in the North West region (than those in North East) respectively. However, the probability of being a joint decision-maker was lower among women whose partners desire more children and those who did not know their partners’ desires.

**Conclusions:**

Women’s age, highest level of education, employment status, wealth index, ethnicity, religion, region of residence and male partners’ desire for children are associated with contraceptive decision making respectively. There is a need for reproductive empowerment interventions in Nigeria that devise effective ways of improving contraceptive decision-making power of partnered women aged 15–24 years, unemployed, in the poorer and richest groups, from the Hausa/Fulani/Kanuri/Beri Beri ethnic group, practising Islam/ other religions, have the same fertility desire as their partners and those who do not know their male partner’s desire for children respectively. Women whose partners desire more children should be empowered to participate effectively in contraceptive decision making.

## Background

Participation in contraceptive decision-making among women has been documented to play important role in boosting contraceptive prevalence [1]. Women who can independently make contraceptive decisions or discuss and reach a joint decision with their male partners that result in contraceptive use are said to have contraceptive decision-making ability [1]. This implies that such women are empowered to make family planning decisions given that studies have linked contraceptive use to female autonomy and empowerment [2–4]. In other words, empowered women are more autonomous or more influential in decision-making in the household which in turn can boost their decision to use contraceptives [5–7].

In the context of marriage, there is a lingering question on factors that determine contraceptive decision-making ability among married women. This area is worth examining because contraceptive decision making can also indicate a married woman’s decision-making ability in her household. Therefore, ability to make contraceptive decision reflects the power dynamics that exist between couples that can shape to an extent, their married reproductive agency.

Unequal power exists between married men and women across cultures in Nigeria due to the reigning patriarchal system that supports men’s dominance over women. Power imbalance prevails in reproductive decision-making such that male partners have greater influence, power, and authority over their female partners on fertility and reproductive matters [3, 8]. Men influence their female partners’ intention to use contraception [8–12]. In the same vein, they are influential in abortion decision-making [13, 14]. Therefore, married women’s ability to make a contraceptive decision on their own (as well as their level of sexual agency) is quite low; many of them would not dare go against their male partners’ disapproval of contraception by overtly practising contraception [8]. This situation hinders many married women from achieving their reproductive needs and puts them at risk of sexual and reproductive health challenges [15]. More so, it can threaten Nigeria’s chances of achieving the sustainable development goal (SDG) 5 which targets gender equality and women empowerment [16].

Women bear the burden/ challenges of pregnancy and would strive to better their reproductive health. In line with this, a woman’s ability to make contraceptive decision to improve her reproductive health as well as the underlying determinants deserve to be examined especially in the context of marriage where partner’s, household factors and other contextual factors can play important roles. Being able to make contraception decision reflects some level of reproductive agency and reproductive empowerment. It is reasonable to assume that women who are empowered to exercise their reproductive rights and make reproductive decision are better positioned to negotiate their contraceptive intentions with their male partners and even make their decision to use contraceptive. Also, such women can negotiate and secure a joint decision with their male partners to use contraceptive. Furthermore, ability to prevent the occurrence of unplanned pregnancy amid spousal violence by deciding to use contraceptive can limit the likelihood of unplanned pregnancy (that may lead to miscarriage due to spousal violence) and undesired fertility. On the other hand, Nigerian men desire more children desire than women [17], and they are more powerful reproductive decision-makers. This makes them highly influential in interspousal discussions on contraception which may result in their disapproval or approval of their female partners’ decision to use contraceptive. Even when they approve of their female partners’ contraceptive decision, they could influence their partner’s choice of method as well as their consistency of use which may not serve their female partners’ interests. Such dominance over reproductive affairs places women at the mercy of their male partners who may decide to sabotage their female partners’ contraceptive use—a form of reproductive coercion [18]. As a result of this, some women resort to practising covert contraception [19].

Despite the dominance of male partners in contraceptive decision-making, some studies have shown that there are powerful factors that tilt contraceptive decision-making power in the favour of married women. For instance, it has been found that women who are educated [20], as well as those employed [20, 21], are more likely to make contraceptive decisions independently or make joint decisions with their male partners. On the other hand, the positive influence of wealth on married women’s household decision-making ability has been established [22, 23]. Given that contraceptive decision-making ability reflects the extent of married women’s decision-making abilities in the household, it is unclear whether wealth plays any role in their contraceptive decision-making abilities.

Men’s absolute control over their female partners’ contraceptive decision-making amid the Nigerian Government’s lack of intention to make policies to ensure gender equality leaves much to be desired. This is because achieving gender equality (in line with the sustainable achieving development goal (SDG) 5) can impact positively the well-being of women. Also, men’s absolute power over contraception can have some negative implications for policymakers and programme managers’ efforts to increase contraceptive uptake, especially the use of modern methods among women to improve their reproductive health (in line with SDG 3). Therefore, it is important to examine the underlying determinants of married women’s ability to make their own contraceptive decisions or make such decisions in conjunction with their male partners rather than their male partners deciding for them.

Based on the foregoing, the overall goal of this study was to examine the underlying determinants of contraceptive decision making among partnered women in Nigeria. Specifically, this study sought to identify the women’s and male partners’ sociodemographic and economic factors associated with contraceptive decision making in Nigeria. Insights from this study can assist the Nigerian government in its commitment and efforts toward reducing the social and gender factors hindering women’s and girls’ agency and autonomy, and access to right-based family planning information and services [24].

## Methods

### Data source

This study analysed the female recode file from the 2018 Nigeria Demographic and Health Survey (DHS) datasets. The DHS is implemented by the National Population Commission (NPC) with the technical support of ICF through the DHS Program [17]. The NDHS data is used to provide demographic and health indicators based on which policymakers and programme managers track, evaluate, and develop public health interventions to improve population health and the health sector [17]. The target population in the 2018 NDHS were women (aged 15–49 years) and men (aged 15–59 years). The survey was implemented using a stratified cluster sampling technique. First, each of the 36 states of the federation was stratified into urban and rural areas (strata). Subsequently, from each stratum, a random selection of enumeration areas (EAs) was done followed by a random selection of eligible Households from each selected EA. All eligible women and men in each selected household were interviewed. A total of 41,821 women were interviewed in the survey.

### Target population

This study targeted only women of reproductive age (15–49 years) who were currently married or living with a man (i.e., partnered women) and currently using a contraceptive method at the time of the survey. Women who were not currently using a contraceptive method were excluded because a woman is expected to be able to state who made the decision that led to her using a contraceptive**.** Hence, a weighted sample of 4,843 women currently married or living with a partner (in-union) and currently using any contraceptive methods was considered in this study.

### Measures

Table 1 describes the variables considered in this study and how each of them was generated. The selection of the explanatory variables was informed by previous studies on contraceptive decision making and contraceptive use [1, 25–29]. The outcome variable is contraceptive decision making. This variable is based on this question posed to the women: “*would you say that using contraception was mainly your decision, your (husband's/partner's) decision, or did you both decide together?”* The response options are mainly respondent (1), mainly husband/partner (2), joint decision (3); and others (6). This was recategorized as women decision makers (1), male partner decision makers (2), and joint decision makers (3). Others (6) were excluded.

**Table 1:** Description of variables, variable labels, and value labels

Sn	Types of Variables	Questions and/or Variable Names in the Dataset	Variable Labels and Values in the Dataset	Variable Labels and Value Labels
**1**	**Outcome variable**			
	Contraceptive Decision-maker	Would you say that using contraception is mainly your decision, mainly your (husband's/partner's) decision, or did you both decide together? (v632a)	Mainly respondent (1) Mainly husband/partner (2) Joint Decision (3) Other (specify) (6)	Women decision makers (1); Male partner decision makers (2); and joint decision makers (3)**.** Others (6) were coded as missing
**2**	**Explanatory variables**			
***2.1***	***Personal socio-demographic and economic characteristics***			
a	Age in 5-year groups (v013)	How old were you at your last birthday?	15–19 years (1); 20–24 years (2); 25–29 years (3); 30–34 years (4); 35–39 years (5); 40–44 years (6) and 45–49 years (7)	15–24 years (1); 25–29 years (2); 30–34 years (3); 35–39 years (4); 40–44 years (5); and 45–49 years (6)
b	Type of marriage	Including yourself, in total, how many wives or live-in partners does he (male partner) have?	No other wives (0),…, 12	Monogamous (0) and polygamous (1–12)
c	Highest educational level	What is the highest level of school you attended: primary, secondary, or higher? (v106)	No education (0); primary (1); secondary (2) and higher (3)	
d	Employment status	Have you done any work in the last 12 months?	Yes (1) and No (2)	Unemployed (1) and Employed (2)
e	Wealth index	v190	Poorest (1); Poorer (2); Middle (3); Richer (4); and Richest (5)	
f	Type of place of residence	v025	Urban (1) and Rural (2)	
g	Ethnicity	What is your ethnic group? (v131)	Ekoi (1); Fulani (2); Hausa (3); …; Igbo (6); Kanuri/Beri Beri (8);…; Yoruba (10); Others (96) and Don’t Know (98)	Hausa/Fulani/Kanuri/Beri Beri (1); Yoruba (2); Igbo (3); and Others (4)
h	Religion	What is your religion? (v130)	Catholic (1); Other Christian (2); Islam (3); Traditionalist (4); and other (96)	Christianity (1) (i.e., any of Catholic and other Christians); and Islam/other (2) (i.e., traditionalist and other)
i	Region	v024	North Central (1); North East (2); North West (3); South East (4); South South (5); and South West (6)	
j	Extent of justification of wife-beating	Beating is justified if wife:goes out without telling husband (v744a), neglects the children (v744b), argues with husband (v744c), refuses to have sex with husband (v744d) and burns the food (v744e)	Yes (1), No (0)	Summed together to generate composite scores ranging from 0 to 5
***2.2***	***Male partners’ socio-demographic and economic characteristics***			
a	Age	How old was your (husband/partner) on his last birthday? (v730)	15, 16. 17, …, 95	Young adult (15–35 years); middle-age (36–55 years); and older adult (56 and above)
b	Education level	What was the highest level of school he attended: primary, secondary, or higher? (v701)	No education (0); Primary (1); Secondary (2); and Higher (3)	
c	Employment status	What is your (husband's/partner's) occupation? That is, what kind of work does he mainly do? (v705)	Did not work (0); professional/technical/managerial (1); clerical (2); …; unskilled manual (9); other (96); and do not know (98)	Unemployed (1) (any of professional/technical/managerial; clerical; …; unskilled manual and other); and Employed (0) (i.e., did not work). Don’t know was coded as missing
d	Partner’s desire for children	Does your (husband/partner) want the same number of children that you want, or does he want more or fewer than you want? (v621)	Both want the same (1); husband wants more (2); husband wants fewer (3); Don’t know (8)	Desire same as partner (1); Partner desires more children (2); Partner desire fewer (3); Don’t know (8)

Women in the age group 15–19 years and 20–24 years were combined to 15–24 years, thus boosting the sample of women in the group because of the very low sample of women in the ages 15–19 and 20–24 years respectively compared to the other older groups. Women aged 15–24 are also regarded as youths (United Nations General Assembly 2001) [30]. Wealth index was computed using principal component analysis by the DHS program. Full details are freely available in the 2018 Nigeria Demographic and Health Survey (NDHS) report. Extent of justification of wife-beating is a composite score (ranging from 0 to 5) computed from five yes or no questions on conditions under which wife-beating is justified. The higher the score the high the extent of justification. The categorization of the ethnicity reflects the three dominant ethnic groups in Nigeria, such as the Hausa/ Fulani/Kanuri/Beri Beri, the Yoruba, and the Igbo ethnic groups [31, 32], while other groups were classified as “others”. Religion was categorized to reflect the two main religions in Nigeria (namely Christianity and Islam), while “others” were combined with Islam due to the low count of women in the “others” group. In recategorizing partners’ age, an earlier method was adopted [33]. This was done to examine whether contraceptive decision-making was hindered among women with middle or older-aged men– which to an extent indicates a higher level of maturity and more exposure to patriarchal culture—compared to those with young men.

### Data analysis

In this study, all the analyses performed were weighted (using the *svyset* command in the Stata (version 14) package [34] using the weighting factors provided in the 2018 Nigeria Demographic and Health Survey (NDHS) data for the females. Weighting was done to adjust all results for sampling and non-response bias. Descriptive analyses of all the variables were presented using frequency and percentage distribution. This was followed by a Chi-square test of independence to examine the association between each sociodemographic and economic factor and contraceptive decision-makers (i.e., the outcome).

Multiple multinomial logistic regression was fitted to examine the relationship between the explanatory variables and the outcome. This model was deemed appropriate for modelling the outcome “contraceptive decision making” because of its polytomous nature [35]. In other words, the outcome variable has three possible outcomes that are not ordered while more than one explanatory variable was considered. Treating the outcome group “male partner decision makers (2)” as the base outcome, the ratio of the probability of women decision makers (1) to male partner decision makers, and the ratio of the probability of joint decision makers (3) to male partner decision makers (2) were estimated, respectively, given the explanatory variables. The Stata *mlogit* command was used to fit the multiple multinomial logistic regression [34]. The multiple multinomial logistic regression model is mathematically expressed as:

$$\frac{p \left(y=1\right)}{p\left(y=2\right)}={e}^{\mathrm{X\beta }(1)}$$

$$\frac{p \left(y=3\right)}{p\left(y=2\right)}={e}^{\mathrm{X\beta }(3)}$$

(y = 1) = The probability of women decision-makers relative to the base outcome (male partner decision makers (P (y = 2)).

*p* (y = 3) = The probability of joint decision-makers relative to the base outcome.

e = Approximately 2.7183.

**X** = Vector of explanatory variables (e.g., women’s age, the highest level of education, etc.)

**β** = Vector of coefficients of each explanatory variable.

The ratios of the probabilities in the above equations are known as the relative risk ratios (RRR) [34]. All the explanatory variables were added to the model to generate the RRRs showing the marginal effect of each explanatory variable while holding other variables constant. More importantly, given that this study analysed complex survey data (i.e. the DHS) of a subsample of all the women interviewed in the survey (i.e., partnered women using any method of contraceptive) the *subpop* command [34] was applied to the Chi-square test (of independence) and the regression model to restrict the analyses to married/ in-union women using any method of contraceptive. This was done to generate accurate point estimates and standard errors in the regression model [36]. The statistical significance of the Chi-square test of independence, the RRRs and the overall regression model was tested against a 5% level of significance. For the RRRs, 95% confidence intervals were computed.

## Results

### Descriptive analysis

#### Sociodemographic and economic characteristics of women and male partners

In Table 2, less than a quarter of women made their own decision to use contraceptives (23%), those whose male partners decided for them constituted about one-tenth (10%), while 67% of women were joint decision makers. More than half of women were less than 35 years old (53%). Most women were in monogamous marriages (82%). More than two-thirds of women had at least secondary education (68%). Most women were employed (83%). More than two-thirds of women were rich (68%) while less than two-thirds resided in urban areas (64%) respectively. More than a quarter of women belonged to the Yoruba ethnic group (29%) while less than a quarter belong to the Igbo group (23%). Less than two-thirds of women were affiliated with Christianity (65%). Less than two-thirds resided in the southern region (63%) of Nigeria. Most women didn’t justify wife beating under any conditions (85%). In terms of male partners’ characteristics, majority of women had male partners above 35 years old (72%). Majority of women stated that their male partners had at least secondary education and were employed respectively (72% and 98% respectively). Concerning male partners’ desire for children, more than half of women said they had the same desire for children (53%).

**Table 2:** Distribution of women and male partners by outcome and explanatory variables

Variables	Frequency	Percentage
**Outcome**		
**Contraceptive Decision Maker**		
Woman only	1,125	23.30
Male partner only	491	10.17
Joint decision	3,213	66.53
**Sociodemographic & Economic Characteristics *Women***		
**Age**		
15–24 years	534	11.03
25–29 years	988	20.40
30–34 years	1,070	22.09
35–39 years	1,129	23.31
40–44 years	728	15.03
45–49 years	394	8.14
**Type of marriage**		
Polygamous	857	17.82
Monogamous	3,953	82.18
**Highest Educational Level**		
No education	674	13.91
Primary education	890	18.37
Secondary education	2,352	48.56
Higher education	928	19.16
**Employment Status**		
Unemployed	836	17.27
Employed	4,007	82.73
**Wealth Index**		
Poorest	262	5.41
Poorer	485	10.01
Middle	817	16.88
Richer	1,412	29.16
Richest	1,867	38.54
**Type of Place of Residence**		
Urban	3,108	64.18
Rural	1,735	35.82
**Ethnicity**		
Hausa/ Fulani/Kanuri/Beri Beri	803	16.58
Yoruba	1,418	29.27
Igbo	1,126	23.26
Others	1,496	30.89
**Religion**		
Christian	3,149	65.02
Islam/other	1,694	34.98
**Region**		
North Central	660	13.64
North East	462	9.54
North West	664	13.70
South East	813	16.80
South South	603	12.46
South West	1,640	33.86
** Extent of justification of wife-beating**		
**0**	4,118	85.03
**1**	188	3.89
**2**	133	2.75
**3**	122	2.52
**4**	96	1.98
**5**	186	3.84
***Male partners***		
**Age**		
Adolescent/ Young Adult (15–35)	1,356	28.00
Middle-aged (36–55)	3,085	63.69
Older adult (56 and above)	403	8.31
**Education Level**		
No education	470	9.81
Primary education	683	14.25
Secondary education	2,355	49.11
Higher education	1,287	26.83
**Employment Status**		
Unemployed	115	2.39
Employed	4,705	97.61
**Partner’s desire for children**		
Desire same as partner	2,518	52.75
Partner desires more children	1,325	27.77
Partner desire fewer	375	7.86
Don’t know	555	11.62

Nigeria Demographic and Health Survey (DHS), 2018; Sample size** = **4,843

### Bivariate analysis

#### Association between personal and male partners’ socio-demographic and economic characteristics and contraceptive decision making

Table 3 shows that all women’s and their male partners’ socio-demographic and economic characteristics except male partners’ employment status were significantly associated with contraceptive decision-making (*p* < 0.05).

**Table 3:** Association between socio-demographic and economic characteristics and contraceptive decision maker

**Sociodemographic & Economic Variables**		**Contraceptive decision maker**			
	**Woman only (%)**	**Male partner only (%)**	**Joint decision (%)**	**χ**^**2**^	***p*-value**
**Age**				4.284	0.001
15–24 years	19.93	17.38	62.69		
25–29 years	18.33	12.48	69.19		
30–34 years	23.82	8.27	67.91		
35–39 years	23.90	8.33	67.77		
40–44 years	27.91	7.98	64.11		
45–49 years	28.58	9.13	62.29		
**Type of marriage**				49.701	0.001
Polygamous	37.95	12.65	49.41		
Monogamous	19.95	9.49	70.56		
**Highest Educational Level**				10.648	0.001
No education	30.80	18.36	50.84		
Primary education	26.58	9.73	63.69		
Secondary education	21.51	8.59	69.90		
Higher education	19.23	8.69	72.08		
**Employment Status**				27.541	0.001
Unemployed	24.13	18.92	56.95		
Employed	23.12	8.36	68.52		
**Wealth Index**				4.336	0.001
Poorest	35.80	11.29	52.91		
Poorer	22.52	15.28	62.20		
Middle	22.88	11.88	65.24		
Richer	25.63	9.20	65.17		
Richest	20.16	8.69	71.15		
**Type of Place of Residence**				3.861	0.022
Urban	22.94	8.83	68.23		
Rural	23.92	12.59	63.49		
**Ethnicity**				9.536	0.001
Hausa/ Fulani/Kanuri/Beri Beri	25.39	19.33	55.28		
Yoruba	23.80	6.62	69.58		
Igbo	21.40	5.83	72.77		
Others	23.12	11.91	64.97		
**Religion**				23.633	0.001
Christianity	20.97	7.86	71.17		
Islam/other	27.61	14.47	57.92		
**Region**				6.734	0.001
North Central	18.64	13.35	68.01		
North East	31.14	20.72	48.14		
North West	21.74	14.11	64.15		
South East	21.43	6.29	72.28		
South-South	21.08	7.36	71.56		
South West	25.33	7.30	67.37		
**Extent of justification of wife-beating**				2.569	0.006
**0**	22.58	9.63	67.78		
**1**	33.05	15.46	51.50		
**2**	28.13	14.59	57.28		
**3**	23.73	10.83	65.44		
**4**	22.39	18.62	58.99		
**5**	25.97	8.88	65.15		
**Age**				5.328	0.001
Adolescent/ Young Adult (15–35)	19.77	12.17	68.06		
Middle-aged (36–55)	23.64	9.43	66.93		
Older adult (56 and above)	32.50	9.17	58.33		
**Education Level**				4.364	0.001
No education	29.29	15.08	55.63		
Primary education	22.95	10.13	66.92		
Secondary education	23.74	8.96	67.30		
Higher education	18.82	10.95	70.23		
**Employment Status**				1.710	0.184
Unemployed	32.08	15.17	52.75		
Employed	23.12	9.98	66.90		
**Partner’s desire for children**				35.133	0.001
Desire same as partner	13.91	8.19	77.90		
Partner desires more children	38.09	11.88	50.03		
Partner desire fewer	21.07	7.69	71.24		
Don’t know	34.13	15.37	50.50		

Nigeria Demographic and Health Survey (DHS), 2018; Sample size** = **4,843; **χ**^**2**^** = Chisquare value**

### Multivariable analysis

#### Relationship between personal and male partner’s socio-demographic and economic characteristics and contraceptive decision making

In Table 4, woman’s age, highest level of education, employment status, wealth index, ethnicity, religion, region, and male partner’s desire for more children were individually associated with contraceptive decision making. The breakdown of the results shows that the probability of women making their own contraceptive decision, relative to the probability of their male partners making the contraceptive decision for them, was twice as higher among women aged 30–34 years (RRR = 2.18; 95%CI = 1.20–3.97; *p* = 0.01), 35–39 years (RRR = 2.06; 95%CI = 1.06–4.03; *p* = 0.03), 40–44 years (RRR = 2.19; 95%CI = 1.08–4.43; *p* = 0.03) and 45–49 years (RRR = 2.74; 95%CI = 1.12–6.71; *p* = 0.03) than women aged 15–24 years respectively. The probability of women being joint decision makers, relative to that of their male partners making the contraceptive decision, was 69%, higher among women aged 30–34 years (RRR = 1.69; 95%CI = 1.05–2.72; *p* = 0.03) than those aged 15–24 respectively.

**Table 4:** Multinomial logistic regression of contraceptive decision making on women’s and male partner’s socio-demographic and economic factors

**Variables**	**Woman only Vs Male Partner**				**Joint decision Vs Male Partner**			
	**RRR**	***p*-value**	**95%CI**		**RRR**	***p*-value**	**95%CI**	
**Personal sociodemographic & economic characteristics**								
**Age (RC = 15–24 years)**								
25–29 years	1.185	0.458	0.756	1.858	1.307	0.174	0.888	1.923
30–34 years	2.181*	0.011	1.200	3.966	1.688*	0.032	1.047	2.723
35–39 years	2.065*	0.033	1.059	4.028	1.598	0.085	0.937	2.725
40–44 years	2.191*	0.029	1.083	4.435	1.568	0.122	0.887	2.773
45–49 years	2.737*	0.028	1.117	6.706	1.915	0.073	0.941	3.895
**Type of marriage (RC = Monogamous)**								
Polygynous	1.426	0.054	0.993	2.047	0.814	0.236	0.579	1.144
**Highest Educational Level (RC = No education)**								
Primary education	1.229	0.390	0.768	1.970	1.395	0.110	0.927	2.097
Secondary education	1.398	0.215	0.823	2.373	1.894*	0.006	1.200	2.988
Higher education	1.345	0.384	0.690	2.622	1.663	0.059	0.980	2.824
**Employment Status (RC = Unemployed)**								
Employed	1.562*	0.015	1.091	2.235	2.103*	0.001	1.537	2.877
**Wealth Index (RC = Poorest)**								
Poorer	0.395*	0.003	0.215	0.727	0.688	0.223	0.376	1.257
Middle	0.475*	0.022	0.251	0.899	0.772	0.391	0.428	1.394
Richer	0.556	0.075	0.292	1.060	0.797	0.487	0.420	1.512
Richest	0.408*	0.012	0.203	0.821	0.741	0.387	0.375	1.462
**Type of Place of Residence (RC = Rural)**								
Urban	1.107	0.581	0.772	1.586	1.123	0.480	0.814	1.549
**Ethnicity (RC = Hausa/Fulani/Kanuri/Beri Beri)**								
Yoruba	2.036*	0.050	1.000	4.147	2.985*	0.002	1.508	5.910
Igbo	2.753*	0.032	1.090	6.949	2.283	0.053	0.991	5.263
Others	1.599	0.119	0.886	2.886	1.325	0.331	0.751	2.338
**Religion (RC = Islam/ others)**								
Christian	1.142	0.557	0.733	1.777	1.465*	0.044	1.010	2.127
**Region (RC = North East)**								
North Central	0.848	0.561	0.486	1.480	1.221	0.416	0.754	1.976
North West	1.564	0.135	0.869	2.813	2.106*	0.007	1.226	3.617
South East	1.296	0.577	0.521	3.226	1.289	0.553	0.557	2.986
South-South	1.749	0.121	0.862	3.550	1.670	0.096	0.913	3.054
South West	1.791	0.115	0.868	3.697	1.246	0.457	0.698	2.224
**Attitude**	1.067	0.201	0.966	1.177	1.073	0.139	0.977	1.179
**Male partner’s sociodemographic & economic characteristics**								
**Age (RC = Adolescent/ Young Adult (15–35))**								
Middle-aged (36–55)	0.839	0.514	0.496	1.420	0.917	0.636	0.640	1.314
Older adult (56 and above)	1.181	0.692	0.517	2.701	0.951	0.876	0.503	1.798
**Education Level (RC = No education)**								
Primary education	0.757	0.338	0.427	1.339	0.689	0.185	0.397	1.196
Secondary education	0.876	0.644	0.499	1.539	0.663	0.107	0.403	1.092
Higher education	0.729	0.332	0.386	1.380	0.634	0.080	0.380	1.056
**Employment Status (RC = Unemployed)**								
Employed	1.097	0.860	0.392	3.073	1.508	0.347	0.641	3.547
**Partner’s desire for children (RC = Desire same)**								
Partner desires more children	2.291*	0.001	1.631	3.218	0.612*	0.001	0.455	0.824
Partner desire fewer	1.716	0.066	0.966	3.050	1.027	0.922	0.606	1.738
Don’t know	1.387	0.187	0.853	2.254	0.440*	0.001	0.295	0.656
	**Woman only Vs Male Partner**	**Joint decision Vs Male Partner**						
	**RRR**	***p*-value**	**95%CI**		**RRR**	***p*-value**	**95%CI**	
**Age (RC = 15–24 years)**								
25–29 years	1.171	0.496	0.743	1.845	1.308	0.173	0.889	1.926
30–34 years	2.151	0.012	1.181	3.918	1.684	0.034	1.041	2.724
35–39 years	2.076	0.033	1.061	4.060	1.605	0.083	0.940	2.740
40–44 years	2.240	0.025	1.105	4.542	1.583	0.113	0.897	2.794
45–49 years	2.794	0.026	1.132	6.900	1.935	0.069	0.949	3.944
**Type of marriage (RC = Monogamous)**								
Polygynous	1.433	0.050	0.999	2.055	0.817	0.245	0.581	1.148
**Highest Educational Level (RC = No education)**								
Primary education	1.244	0.363	0.777	1.993	1.402	0.105	0.932	2.108
Secondary education	1.406	0.210	0.825	2.394	1.902	0.006	1.204	3.003
Higher education	1.350	0.383	0.688	2.646	1.662	0.062	0.975	2.832
**Employment Status (RC = Unemployed)**								
Employed	1.579	0.013	1.102	2.261	2.110	0.000	1.543	2.887
**Wealth Index (RC = Poorest)**								
Poorer	0.400	0.003	0.218	0.735	0.690	0.228	0.378	1.261
Middle	0.476	0.023	0.251	0.902	0.774	0.397	0.429	1.399
Richer	0.559	0.079	0.292	1.069	0.797	0.486	0.420	1.511
Richest	0.410	0.013	0.204	0.826	0.742	0.390	0.376	1.465
**Type of Place of Residence (RC = Rural)**								
Urban	1.092	0.630	0.763	1.563	1.113	0.512	0.807	1.535
**Ethnicity (RC = Hausa/Fulani/Kanuri/Beri Beri)**								
Yoruba	1.863	0.090	0.907	3.826	2.882	0.003	1.443	5.756
Igbo	2.501	0.050	0.998	6.266	2.181	0.067	0.946	5.029
Others	1.417	0.242	0.790	2.544	1.252	0.438	0.709	2.211
**Religion (RC = Islam/ others)**								
Christian	1.183	0.454	0.762	1.838	1.500	0.030	1.039	2.166
**Region (North East)**								
North Central	0.884	0.660	0.509	1.534	1.243	0.366	0.775	1.994
North West	1.538	0.154	0.850	2.781	2.090	0.009	1.204	3.628
South East	1.319	0.550	0.532	3.270	1.303	0.535	0.564	3.008
South-South	1.785	0.107	0.882	3.609	1.689	0.084	0.931	3.065
South West	1.865	0.091	0.905	3.843	1.264	0.425	0.711	2.248
**Partner’s desire for children (RC = Desire same)**								
Partner desires more children	2.275	0.000	1.619	3.196	0.610	0.001	0.453	0.820
Partner desire fewer	1.706	0.068	0.960	3.030	1.025	0.927	0.605	1.737
Don’t know	1.373	0.202	0.844	2.233	0.439	0.000	0.294	0.656
**Attitude**	1.063	0.227	0.963	1.173	1.072	0.147	0.976	1.177
**Control**	1.100	0.171	0.960	1.261	1.019	0.775	0.898	1.156
**Age (RC = Adolescent/ Young Adult (15–35))**								
Middle-aged (36–55)	0.842	0.519	0.499	1.421	0.914	0.624	0.639	1.309
Older adult (56 and above)	1.165	0.717	0.508	2.671	0.941	0.851	0.497	1.779
**Education Level (RC = No education)**								
Primary education	0.768	0.363	0.435	1.356	0.695	0.194	0.401	1.204
Secondary education	0.884	0.667	0.504	1.551	0.668	0.111	0.406	1.098
Higher education	0.744	0.364	0.393	1.409	0.642	0.087	0.386	1.067
**Employment Status (RC = Unemployed)**								
Employed	1.071	0.895	0.383	2.998	1.498	0.354	0.637	3.519

*Base outcome* Male partner only, *RRR* Relative Risk Ratio, *CI* Confidence interval; *p < 0.05; *RC* Reference Category; Likelihood ratio χ^2^ (68) = 736.68; *p* = 0.001

The probability of women being joint decision makers, relative to the probability of their male partners making the contraceptive decision, was 89% higher among women who had secondary education (RRR = 1.89; 95%CI = 1.20–2.99; *p* = 0.01) than their uneducated counterparts respectively. For employment status, the probability of women making their own contraceptive decision, relative to the probability of their male partners making the contraceptive decision, was 56% higher among women who were employed (RRR = 1.56; 95%CI = 1.09–2.23; *p* = 0.01) than the unemployed. Among employed women (compared to the unemployed) the probability of women being joint decision makers (relative to that of their male partners making the contraceptive decision, was two times higher (RRR = 2.10; 95%CI = 1.54–2.88; *p* = 0.01).

In terms of wealth index, the probability of women making their own contraceptive decision (relative to the probability of their male partners making the contraceptive decision for them) was 61%, 53% and 59% lower among the women in the poorer (RRR = 0.39; 95%CI = 0.21–0.73; *p* = 0.01), middle (RRR = 0.47; 95%CI = 0.25–0.90; *p* = 0.02) and richest groups (RRR = 0.41; 95%CI = 0.20–0.82; *p* = 0.01) than the poorest women respectively.

As regards ethnicity, compared to women from the Hausa/Fulani/Kanuri/Beri Beri ethnic group, the probability of being the sole decision-maker (than being the male partner’s decision) was two times higher among the Yorubas (RRR = 2.04; 95%CI = 1.00–4.15; *p* = 0.05) and Igbos (RRR = 2.75; 95%CI = 1.09–6.95; *p* = 0.03) respectively. Besides, the probability of being joint decision makers (relative to the probability of their male partners making the contraceptive decision) was twice as higher among the Yorubas (RRR = 2.98; 95%CI = 1.51–5.91; *p* = 0.01).

Compared to the Muslims/ others, the probability of women being joint decision makers (relative to the probability of their male partners making the contraceptive decision for them) was 46% higher among the Christians (RRR = 1.46; 95%CI = 1.01–2.13; *p* = 0.04). The probability of women being joint decision makers (relative to the probability of their male partners making the contraceptive decision for them) was two times higher among women in the North West (RRR = 2.11; 95%CI = 1.23–3.62; *p* = 0.01) than women in the North-East region. Lastly, women who stated that partners desired more children, compared to those who stated that they wanted the same, were two times more likely to have solely made their contraceptive decision, than state that it was their partner’s decision (RRR = 2.29; 95%CI = 1.62–3.22; *p* = 0.01). In contrast, the same group of women, alongside those who stated that they did not know about their male partners’ desire for children, were 39% and 56% less likely to be joint decision-makers (RRR = 0.61; 95%CI = 0.45–0.82; *p* = 0.01 and RRR = 0.44; 95%CI = 0.29–0.66; *p* = 0.01, respectively) respectively than state that it was their male partners’ decision.

## Discussion

This study contributed to the existing knowledge on the nexus between women empowerment and their use of family planning by specifically examining women’s and their male partners’ socio-demographic and economic factors associated with the likelihood of women making their own contraceptive decision or jointly deciding with their male partners rather than their male partners deciding for them.

Overall, there were more women (contraceptive) decision-makers than male partner decision-makers. This was also observed across women’s and their male partners’ sociodemographic and economic attributes except for male partners’ employment status. However, joint decision contraceptive decision-makers constituted the largest proportion among the women. This is consistent with earlier studies in Nigeria [11], sub-Saharan Africa [28], and the United States of America [37].

In this study, women’s age was associated with contraceptive decision making. The likelihood of women making their own contraceptive decision was higher among older women (aged 30–34, 40-44 years and 45–49 years) than among the youngest women (aged 15–24 years). This support the findings from a study conducted in Ethiopia where women aged 15–24 were more likely to dominate contraceptive decision [20]. In contrast, a study in South-East Ethiopia revealed that married women aged 18–20 years (compared to their oldest counterparts aged 35 years and above) were more powerful family planning decision-makers [38]; although the inconsistencies in their findings and this study’s may be attributed to difference in the age grouping. Besides the likelihood of being joint decision-makers was higher among women aged 30–34 years. These findings (in this study) suggest that as women advance in age, they become more mature and able to make a contraceptive decision or engage in contraceptive discussion with their male partners that leads to a joint contraceptive decision.

Women who had secondary education (compared to the uneducated group) were more likely to be joint decision makers (than state that the decision was made by their male partners). This is unlike the findings from Honduras where women with primary education were more likely to state that their male partners were responsible for their family planning decision [39]. Yet, it has also shown that education could enable women to secure a joint contraceptive decision [20]. This is because education is a source of empowerment that can improve a woman’s ability to negotiate her reproductive intention with her male partner better and make reproductive decision apart from being able to gather useful information and interact with the outside world [40].

Women who were employed were more likely to have made their contraceptive decision and be joint decision makers respectively than state that the decision was made by their male partners. This result supports the previous findings that women who are employed are more likely to decide independently to use contraceptive [21]. Just like education, engaging in economic activities can improve a married/in-union woman’s social-economic status and her prestige (through access to workplace leadership positions). This can enhance her ability to communicate her contraceptive desires with her male partner and increase her chances of securing a joint contraceptive decision.

This study revealed that the likelihood of being solely responsible for contraceptive decision (than being the male partner’s decision) was lower among women in the poorer, middle, and richest categories (than those in the poorest group) respectively. Wealth index in this study is a household characteristic. Most partnered men are breadwinners and are often solely responsible for the purchase of assets in the household, this increases their tendency to dominate major decisions including their female partners’ reproductive decisions. On the other hand, women’s wealth should increase their odds of making household decision-making because wealthy women are not only socio-economically empowered, they also command attention from their society and can influence their household decisions [22, 23]. Therefore, it was expected that wealth should improve women’s ability to make or participate in contraceptive decision-making just like in a similar recent study on sub-Saharan Africa (SSA) [28]. However, the result was contrary to expectations and further implies that there need for state-level analyses of women’s contraceptive decision-making because analyses at the SSA level may conceal interesting state-specific evidence that can inform state-specific interventions. These surprising results may be explained by a study in the southeast region of Nigeria, where the interviewed women stated that ownership of properties by a married woman does not necessarily grant her reproductive decision-making power [41]. This view is underpinned by the patriarchal system (across ethnic groups in Nigeria) which promotes male partners’ dominance in reproductive decision-making, and which is difficult to challenge even if their female partners are wealthy.

While the likelihood of being the main decision maker (rather than being the male partner) was higher among women from Yoruba and Igbo ethnic groups respectively compared to the Hausas/Fulanis/Kanuris/Beri Beris, the probability of being a joint decision maker, relative to being the male partner's decision, was higher among women from the Yoruba ethnic groups (than women from the Hausa/Fulani group) respectively. Hausas/Fulanis/Kanuris?Beri Beris women are less empowered in making decisions in their households because of the patriarchal system they are subject to and their practice of the Sharia [42]. This may have contributed to their inability to initiate interspousal discussion on contraceptive let alone secure their male partners’ support to use contraception. More importantly, the desire for more children is still high among the Hausas/ Fulanis/ Kanuris/ Beri Beris and this has consistently contributed to high fertility among them. Hence, it is difficult for Hausa/ Fulani/ Kanuri/ Beri Beri women to present their contraceptive desires and intentions to their male partners in the hope of securing their support. The high literacy, education, and socio-economic status of Yoruba women (unlike Hausa/ Fulani/ Kanuri/ Beri Beri women) may have been the reason behind their higher likelihood of being sole decision makers and joint decision makers respectively. The same reason also applies to Igbo women who have a higher likelihood of being the main contraceptive decision-makers. Besides, evidence has shown that Yoruba women are more likely to achieve their reproductive desires through interspousal discussion on their contraceptive intentions [8].

Concerning religion, it was found that Christians, relative to other women affiliated with Islam or other religions, were more likely to be the joint contraceptive decision-makers. Being joint decision-makers may be due to the flexibility and less conservativeness of Christians. Although, the religion of male partners may also play a significant role. Nevertheless, Christian women tend to enjoy more rights and freedom than their Muslim counterparts [10].

Again, the level of empowerment and autonomy among women in northern Nigeria is low [5], thus affecting their decision-making ability in their households [43], and their ability to participate in reproductive decision-making [22]. This cannot be divorced from women’s low uptake of contraceptive in the region. Therefore, logically, the likelihood of being joint decision-makers among partnered women across the north was expected to reduce. Surprisingly, this study revealed that the likelihood of being joint decision-makers was higher among women in the North-West region (than their counterparts in the North East region). This suggests that being in the northern region does not absolutely hinder a woman’s ability to negotiate and reach a joint decision with her partner to use contraceptive. This result creates an impression (that future studies can explore) that the North West region may have some unique contextual characteristics (not considered in this study) that promote women’s contraceptive decision-making ability. Also, these findings question the tendency to conclude that issues surrounding contraception intention and use are the same across the northern region.

Women who stated that their male partners desired more children were more likely to be contraceptive decision-makers (relative to their male partners) than women who had the same desires as their partners. This group of women may be covertly using contraception. This is because it has been observed that women in Nigeria often use contraception covertly for health reasons [8, 44–46], when they find it difficult to engage in interspousal communication with their male partners on contraception, if their male partners object to contraception, or their male partners desire many children [19]. On the other hand, such women who stated that their male partners desired more children were less likely to be joint contraceptive decision-makers (relative to their male partner’s decision) than those who had the same desire as their male partners. Male partners who are pronatalist are more likely to make contraceptive decisions for their female partners. Given their domineering status in couples’ reproductive matters, they may also end up sabotaging their female partners’ contraceptive decisions. Women who did not know their partners’ desires were less likely to be joint decision-makers (relative to the likelihood of their partners making the decision) compared to women who had the same desire as their male partners. Likely, such women do not discuss their fertility desires with their male partners to know their partners’ fertility desires. Lack of intentional interspousal communication can rob such women of the opportunity to initiate contraceptive discussions that can end up favouring their desire to use contraception.

The study is limited in terms of its lack of internal validity because information on the explanatory and outcome variables considered was collected concurrently during the survey, therefore it is difficult to determine the temporal precedence between the explanatory and outcome variables. Other factors such as socio-cultural factors (cultural and religious beliefs and gender norms) that may determine the likelihood of contraceptive decision-makers were not considered in this study because such information was not covered in the DHS data. The sample was not large enough to permit the estimation of the likelihood of the outcome among women from each of the “other” ethnic groups. Nevertheless, given that the DHS data is representative at national and state levels, coupled with the application of complex survey weights, this study presents a reliable picture of the sociodemographic and economic correlates of contraceptive decision-makers among partnered women in Nigeria.

## Conclusion

This study concludes that women’s age, highest level of education, employment status, wealth index, ethnicity, religion, region of residence and male partner’s desire for children are correlates of contraceptive decision making respectively. Therefore, there is a need for reproductive empowerment interventions in Nigeria that devise effective ways of improving contraceptive decision-making power of partnered women aged 15–24 years, unemployed, in the poorer and richest groups, from the Hausa/Fulani/Kanuri/Beri Beri ethnic group, practising Islam/ other religions, have the same fertility desire as their partner and those who do not know their male partner’s desire for children respectively. Women whose partners desire more children should be empowered to participate effectively in contraceptive decision making.

## Data Availability

The NDHS data analysed in this study was obtained with permission from the DHS program. The data is publicly available and accessible through this direct (website) link: https://dhsprogram.com/data/available-datasets.cfm

## Abbreviations

NDHS: Nigeria Demographic and Health Survey
RRR: Relative Risk Ratio
SDG: Sustainable Development Goal
